# Towards a human-rights-based framework for assessing psychiatric intervention for children and young people

**DOI:** 10.1192/bjo.2024.856

**Published:** 2025-03-14

**Authors:** Jill Stavert, John Watts, Ulrike M. E. Schulze, Anja Malmendier-Muehlschegel

**Affiliations:** School of Health and Social Care, Edinburgh Napier University, Edinburgh, UK; Department of Adolescent Psychiatry, North East London NHS Foundation Trust, London, UK; Zentrum für Psychiatrie Calw – Klinikum Nordschwarzwald, Calw-Hirsau, Germany; Department of Child and Adolescent Psychiatry, Psychosomatics and Psychotherapy, University of Ulm, Ulm, Germany

**Keywords:** Children and young persons, psychiatric interventions, capacity and competence, human rights, common frameworks

## Abstract

Decisions around psychiatric interventions for children and young people involve balancing respect for the child’s wishes, the need to provide benefit and relevant risk factors. We recommend establishing a framework for assessment of interventions for children with mental disabilities, using a human-rights-based approach that can be applied across jurisdictions, alongside national laws.


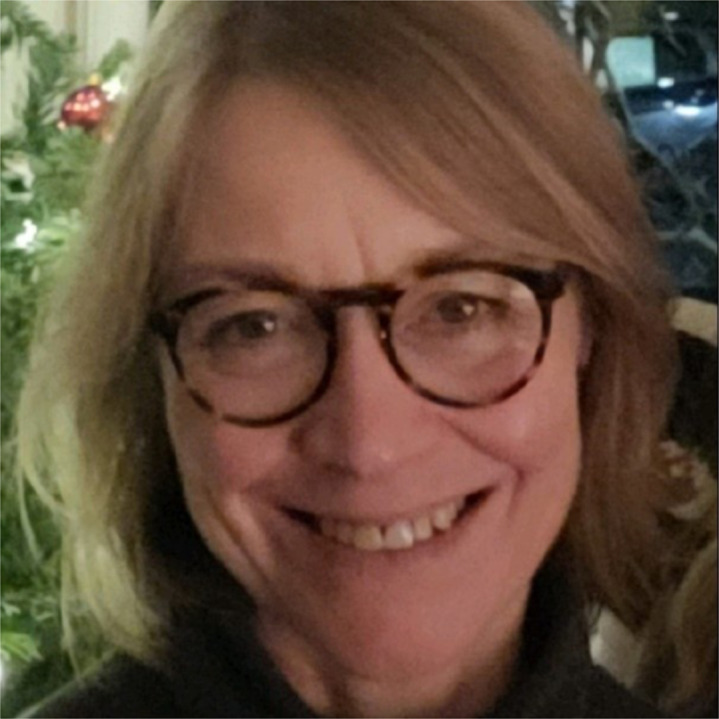
As with adults, decisions around the necessity and nature of psychiatric interventions for minors (children) invariably involve balancing respect for the child’s wishes, the need to provide a benefit for the child and relevant risk factors. This is further complicated by the developing capacity and capabilities of children.

The authors have worked in three different jurisdictions in Europe and during the course of clinical discussions realised that practice varies greatly between similar countries, despite the need to adhere to a universal international legal framework, and despite treating patients with similar presentations and needs. Variations exist in the threshold for hospital admission, and in the role of parental oversight and permission and the limits of this. Major differences include the level of autonomy accorded to a young person and how decision competence is assessed. Across the board, there is a general lack of guidance and advice regarding supported decision-making, including the role, if any, of parents in this. Our aim in this editorial was to summarise relevant human rights standards to encourage an international discussion among mental health practitioners working with minors with mental health conditions (we use this term to encompass patients with mental illnesses and disorders generally amenable to in-patient treatment) to arrive at a more uniform and patient-centred approach.

Given that these issues are relevant to all countries, we suggest that it would be helpful to clinicians to establish a set of common principles or a framework for assessing interventions relating to children with psychiatric disorders, which could be applied across jurisdictions alongside relevant national laws. We consider that this could be achieved through a human-rights-based approach that is informed by the European Convention on Human Rights (ECHR) where European countries are concerned^1^ and the Convention on the Rights of the Child (CRC)^2^ and Convention on the Rights of Persons with Disabilities (CRPD) for all countries.^3^ These treaties identify rights that apply to the psychiatric treatment of children, and most states are parties to such treaties. This means that they are obliged to ensure that national health and social bodies respect these rights, and failure to do so may result in international condemnation by the bodies responsible for monitoring the treaties and often legal action nationally by or on behalf of people whose rights are not respected.

## Human rights standards

When designing any human-rights-based framework for children, one initially looks to the CRC, which applies to children up to 18 years old. The ECHR and CRPD are also applicable and apply to all ages. Many CRC rights, while interpreting such rights uniquely in the context of children, broadly mirror those set out in the ECHR and/or CRPD. Examples particularly relevant to mental healthcare and treatment include the rights to life, liberty, autonomy (including the exercise of legal capacity, and respect for private and family life), dignity and freedom from cruel, inhuman or degrading treatment or punishment, and for such rights to be enjoyed by the child without discrimination associated with their diagnosis.

Importantly, article 3 of the CRC and article 7 of the CRPD both make it clear that in any decisions or actions concerning children, the child’s best interests shall be a primary consideration. Similarly, articles 19 and 23 of the CRC and articles 16 and 19 of the CRPD both identify rights to be free from abuse and violence and to participate in one’s community. They also make it clear that the whole range of socioeconomic rights apply to children with mental disabilities, for instance, the right to the enjoyment of the highest attainable standard of health (article 24 of the CRC and article 25 of the CRPD), thereby recognising the reality that a child’s psychiatric needs should be considered in the wider context, including other needs.

The rights identified in each of these treaties must be read alongside each other when giving effect to such rights. In many cases, this is reasonably straightforward and can be reflected in a framework. However, it will be necessary to decide where the guiding principles sit in relation to the more radical interpretations of CRPD rights.

The CRPD’s monitoring body, the Committee on the Rights of Persons with Disabilities, for example, interprets the non-discriminatory exercise of legal capacity as only being achievable through abolishing non-consensual interventions and psychiatric detention. It considers that such measures are often determined on the basis of mental capacity assessments and ‘best interests’ decisions (which it regards as subjective and biased) and where a full supported decision-making regime should be implemented instead.^4^ This interpretation is not without controversy, and no state has yet implemented the committee’s direction in its entirety. However, it will be necessary to determine whether the guiding principles allow for non-consensual interventions, whether mental capacity assessment would determine this, and the role, if any, and nature of supported decision-making for children in this context.

## Considerations for a framework

If it is accepted that non-consensual psychiatric interventions is required for children, albeit as a last resort, then the proposed guiding principles will need to consider the following:

- Articles 5 and 8 of ECHR case law state that psychiatric detention is only permissible (i) where the law permits this, there is a reliable medical diagnosis and such deprivation of liberty is necessary, proportionate and in an appropriate facility^5^; (ii) there is a practical and effective means by which to challenge the legality of such detention and to have it regularly reviewed,^6^ and the person must be discharged when detention is no longer a proportionate measure; and (iii) a lawful detention does not automatically authorise non-consensual treatment, which must be separately determined according to its necessity and proportionality.^7^ Article 25 of the CRC also specifically set out the right of a child placed by authorities in a psychiatric setting ‘to a periodic review of the treatment provided to the child and all other circumstances relevant to his or her placement’.
- If mental capacity (or competency) assessments are a criterion for non-consensual treatment, then one should note that article 12 of the CRPD goes further than the ECHR in that it requires that persons with mental disabilities enjoy legal capacity on an equal basis to others, with supported decision-making being available to overcome any decision-making challenges to ensure this happens. In relation to children, article 7 of the CRPD states:
  ‘States Parties shall ensure that children with disabilities have the right to express their views freely on all matters affecting them, their views being given due weight in accordance with their age and maturity, on an equal basis with other children, and to be provided with disability and age-appropriate assistance to realize that right.’

This more or less repeats article 12^1^ of the CRC, which states^2^ that a child shall be provided the opportunity to be heard in any judicial and administrative proceedings affecting them, either directly or through representation in a manner consistent with the state’s procedural legal rules. Moreover, article 5 of the CRC recognises the need to respect the responsibilities, rights and duties of parents and, where applicable, extended family and community.

The situation of children is unique in that the persons with parental responsibility can also make decisions about the healthcare of the child; in fact, substitute decision-making is often the norm in younger children, as long as the decisions do not conflict with the best interest of the child. However, as children mature and become increasingly competent with respect to decision-making, a tension between parental decision-making powers and the child’s autonomy can arise.

A parent may be able to support their child to decide, and give consent, but this would only amount to supported decision-making, in other words, demonstrating the child’s genuine will and preferences, where it can be shown there was no undue pressure or a conflict of interest relating to the parent(s). An attempt to navigate this complicated area has been made in England and Wales with the concept of the Scope of Parental Responsibility^8^ in the Code of Practice for the Mental Health Act. This concept includes consideration of the maturity of the child and weighing that against the rights of the parent. It also includes some judging of whether the decision is one that society would deem reasonable for a parent to make. The concept is difficult to standardise, and it is not without its critics.^9^

A human-rights-compliant approach in our suggested framework would therefore importantly need to include determination of:

- whether the child, absent the diagnosis, is considered to have the age and maturity to form a valid decision/opinion; and, if so,
- whether those with parental responsibility can support such decision-making to arrive at the child’s genuine wishes, or whether additional forms of supported decision-making (e.g. advocacy, speech and language therapy, trusted peers and professionals, children’s representative groups) need to be considered to ascertain the genuineness of the child’s decision.

The authors strongly believe that there is a need for discussion exploring suitable frameworks, both from clinical and legal perspectives. We intend to explore these proposals in more detail, and we welcome any feedback or criticism from clinicians, lawyers, academics and children’s representative organisations.

## Data Availability

Data availability is not applicable to this article as no new data were created or analysed in this study.
